# Myélome à Ig D Kappa révélé par des lombosciatiques bilatérales

**DOI:** 10.11604/pamj.2014.18.314.5050

**Published:** 2014-08-21

**Authors:** Wafa Chebbi, Olfa Berriche

**Affiliations:** 1 Service de Médecine Interne, CHU Taher Sfar Mahdia, 5100 Mahdia, Tunisie

**Keywords:** Myélome, IgD Kappa, lombosciatiques bilatérales, Myeloma, IgD Kappa, bilateral sciatica

## Image en medicine

Le myélome multiple à Ig D est une entité rare (1 à 3% des myélomes multiples), caractérisée par sa sévérité clinique et son mauvais pronostic. Le sous-type à Ig D Kappa n'en représente que 10 à 30%. Nous rapportons l'observation d'un patient âgé de 70 ans, sans antécédents pathologiques, hospitalisé pour des lombosciatiques L3 bilatérales, hyperalgiques, d'allure inflammatoire et rebelles aux antalgiques usuels, associées à une altération de l’état général. L'examen trouvait un patient en mauvais état général, apyrétique et une pâleur cutanéo-muqueuse. L'examen neurologique était normal. Il n'y avait pas d'hépatomégalie ni de splénomégalie ni d'adénopathie palpable. Le bilan biologique montrait une vitesse de sédimentation à 90 mm à la première heure, une protéine C-réactive à 12 mg/L et une anémie normochrome normocytaire arégénerative à 8 g/dl d'hémoglobuline. L’électrophorèse des protéines a montré une hypoalbuminémie à 24 g/l, une hypergammaglobulinémie sans pic monoclonal. L'immunoélectrophorèse a montré une discrète bande gamma à Ig D Kappa dans le sang et une bande homogène Kappa liée et libre dans les urines. Il n'y avait pas de protéinurie de Bence- Jones. La calcémie corrigée était à 3 mmol/l. La créatinémie était à 183 µmol/l. Le myélogramme montrait une plasmocytose médullaire à 35% faite de plasmocytes dystrophiques. La recherche d'amylose par biopsie des glandes salivaires accessoires était négative. Les radiographies du rachis lombaire montraient des signes des lésions ostéolytiques avec tassements vertébraux. La tomodensitométrie du rachis montrait une atteinte osseuse lytique multifocale intéressant la totalité des vertèbres lombaires mais touchant son maximum au niveau de L3, où elle s'accompagnait d'une épidurite. Une polychimiothérapie associée aux biphosphonates était instaurée avec amélioration des douleurs lombaires et de l'insuffisance rénale.

**Figure 1:**
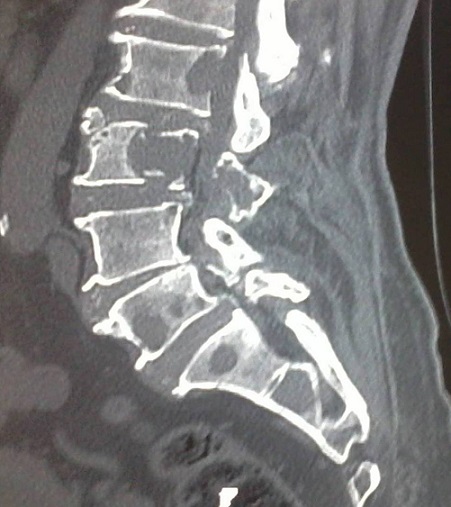
TDM du rachis en coupe sagittale: atteinte osseuse lytique multifocale des vertèbres lombaires avec un maximum au niveau de L3 et une épidurite

